# Impact of benzodiazepine consumption reduction on future burden of dementia

**DOI:** 10.1038/s41598-020-71482-0

**Published:** 2020-09-04

**Authors:** Hélène Jacqmin-Gadda, Florian Guillet, Clément Mathieu, Catherine Helmer, Antoine Pariente, Pierre Joly

**Affiliations:** 1 INSERM, ISPED, Bordeaux Population Health Research Center, UMR 1219, Univ. Bordeaux, Bordeaux, France; 2 Pole de Santé Publique, Service de Pharmacologie Médicale, CHU de Bordeaux, 33000 Bordeaux, France

**Keywords:** Epidemiology, Computational science, Statistics

## Abstract

Dementia is a major public health issue worldwide and chronic use of benzodiazepine, which is very frequent in northern countries, was found to be a risk factor of dementia. This work aims at evaluating the impact of a reduction in chronic use of benzodiazepine on the future burden of dementia in France. Using estimations of dementia incidence and of benzodiazepine use and nation-wide projections of mortality and population sizes, a Monte Carlo approach based on an illness-death model provided projections of several indicators of dementia burden. With no change in benzodiazepine consumption, the prevalence of dementia between age 65 and 99 in France in 2040 was estimated at 2.16 millions (95% confidence interval (CI) 1.93–2.38), with a life expectancy without dementia at 65 years equal to 25.0 years (24.7–25.3) for women and 23.8 years (23.5–24.2) for men. Assuming a disappearance of chronic use of benzodiazepine in 2020, the prevalence would be reduced by about 6.6% in 2040 and the life expectancy without dementia would increase by 0.99 (0.93–1.06) year among women and 0.56 (0.50–0.62) among men. To conclude, a modest but significant reduction in future dementia burden could be obtained by applying current recommendation for duration of benzodiazepine use.

## Introduction

Dementia, whose Alzheimer’s disease is the main cause, is a very frequent disease in older adults throughout the world. Age being the main known risk factor of dementia, the number of cases is expected to increase in the future due to the aging of the population^1^. As dementia progressively leads to complete loss of autonomy requiring a permanent support, this disease represents a tremendous social and economic cost for our societies^2^. Apart from genetic and socio-demographic factors such as gender and educational level, some modifiable factors such as vascular risk factors^3^ or drugs consumption^4^ are suspected to be associated with the risk of dementia. Among them, several studies have shown a higher risk of dementia among benzodiazepine (BZD) users^5–13^. While conflicting results were recently published^14,15^, these findings were confirmed in several meta-analyses^16–18^ and Billioti et al.^19^ demonstrated the plausibility of a causal relationship.

BZD are mainly used as anxiolytic and hypnotic with a recommended duration of treatment of less than 4 weeks in many countries. In France, although the maximum recommended duration of prescription is 4 weeks as hypnotic and 12 weeks as anxiolytics, the duration of use exceeds 3 months for more than 50% of patients. Chronic use of BZD is a major public health concern in most developped countries^20–22^, especially in the older adults who are more likely to have side effects^23^ owing to polymedication and physiological changes with aging. Recommendations against chronic use of BZD are regularly published without any clear impact on the consumption, while applying the current recommendations could dramatically reduce this use. Quantifying the possible impact of such a reduction on the future burden of dementia could strengthen these public health recommendations.

Projections of future burden of dementia may be based on forward calculations or simulations using current estimates of dementia incidence and population projections. To account for differential mortality between demented and non-demented and for the impact of intervention scenarios on mortality trend, projections must rely on multi-state models including transitions to death^24–26^. When the mortality among non-demented was considered as equal to the general mortality^27–29^, the future prevalence was underestimated^24^.

Few studies have evaluated the impact of change in risk factor distributions on future burden of dementia. Some authors evaluated the impact of reduction of prevalence of high blood pressure (HBP) in the population on 20-year projections for dementia prevalence^30^ and various other measures of the disease burden (including life expectancies with and without the disease and lifelong probabilities of the disease)^31^. The above methods, based on analytic computations of epidemiological indicators in continuous time, assumed that the risk factor was acquired at mid-life. Specifically, it was considered that HBP arising later in life had no impact on dementia risk as suggested by previous studies^32,33^. This hypothesis may be debatable for HBP but, for BZD, it is clearly inconsistent with the literature which highlighted an increased risk of dementia associated with chronic use of BZD after 65 years old.

Assuming a causal effect of BZD on dementia risk, the objective of this work is to evaluate the potential impact of a decline in chronic BZD use in the older adults on the future burden of dementia in France, appreciated through several epidemiological indicators (prevalence, lifetime risk, life expectation without dementia,…). To account for the impact of BZD initiated after 65 years old, we propose a Monte Carlo approach in discrete time based on an illness-death model.

## Methods

### Data sources

Three sources of data were used. Incidence of dementia and relative risk of death among demented subjects were estimated from the Paquid cohort. Paquid is a cohort of 3,777 subjects aged 65 years or over at inclusion representative from two French departments^34^. Subjects randomly selected from the electoral rolls who agreed to participate were interviewed at home by trained neuropsychologists at baseline in 1989 and subsequently every two or three years over 27 years. Diagnosis of dementia was assessed using DSM IIIR criteria in a two-phase procedure including a screening by the neuropsychologist and a clinical examination at home by a neurologist. Vital status and exact date of death were collected all along the follow-up. Mortality in the Paquid cohort was shown to be very close from national mortality rate in France for the same period^24^.

Prevalence and incidence of chronic use of BZD from age 65 were estimated from the Echantillon Généraliste des Bénéficiaires (EGB) using data from 2010 to 2015. EGB was established in 2005. This is a 1/97th nationwide representative random sample of the French health insurance beneficiaries whether they are receiving healthcare or not^35^. In France, every citizen is covered by health insurance and EGB includes most insurance systems except a few systems for civil servants and student. Participants are followed until their death, emigration or insurance cessation while newly insured patients are added on a quarterly basis. This database includes age and gender and information on all out-hospital reimbursements of drugs for more than 600,000 subjects representative of the French population. In this work, relying on reimbursement data, chronic use of BZD was defined as 6 months of continuous use as in most published studies about the association between BZD use and dementia^7,10,36^.

Finally the population size by gender at age 65 and the estimations (till 2015) and projections (from 2016) for the overall mortality rates by age and gender were provided by the French National Institute of Statistics (INSEE)^37^, for each year from 1950 to 2070.

### “Illness-death” model

The method relies on a non-homogeneous Markov illness-death model depicted in Supplementary Fig S1 online where the three-states are “non-demented”, “demented” and “dead”. The transition intensities between the states that represent respectively the incidence of dementia ($\alpha_{01}$), the mortality of healthy subjects ($\alpha_{02}$) and the mortality of demented subjects ($\alpha_{12}$) were modeled by the following proportional intensity models:

1 $$ \alpha_{01} \left( {a,t,z\left( {a,t} \right)} \right) = \alpha_{01}^{0} \left( {a,t} \right)\theta_{01} \left( a \right)^{{z\left( {a,t} \right)}} ; $$

2 $$ \alpha_{02} \left( {a,t,z\left( {a,t} \right)} \right) = \alpha_{02}^{0} \left( {a,t} \right)\theta_{02} \left( a \right)^{z(a,t)} ; $$

3 $$ \alpha_{12} \left( {a,t,z\left( {a,t} \right)} \right) = \alpha_{12}^{0} \left( {a,t} \right)\theta_{12} \left( a \right)^{{z\left( {a,t} \right)}} = \alpha_{02}^{0} \left( {a,t} \right)RR\left( a \right)\theta_{12} \left( a \right)^{{z\left( {a,t} \right)}} ; $$

where $a$ is the age, $t$ is the calendar year and $z\left( {a,t} \right)$ is the time and age dependent BZD exposure: $z\left( {a,t} \right)$ equals 1 if the subject is exposed at time *t* and age *a* and 0 if not. Dementia incidence and mortality rates depend on both age and calendar time. The mortality among demented is proportional to the mortality of healthy subjects with a relative risk depending on age $(RR(a))$ while $\theta_{01} \left( a \right)$, $\theta_{02} \left( a \right)$ and $\theta_{12} \left( a \right)$ are the relative risks associated with the exposure to BZD, respectively for dementia, mortality among healthy subjects and mortality among demented subjects.

### Estimations of the incidence and prevalence at age 65 of chronic use of BZD

Subjects from EGB were eligible for this study if they: (i) had been continuously affiliated to the main French Health Insurance scheme (so-called General scheme) at least from 2009/01/01 to 2010/02/01, (ii) had a complete follow-up from 2009 until death, date of general scheme exit, or until 31 December 2015, whichever came first, (iii) were aged 65 or over on 2010/01/01. Subjects were considered as chronic BZD users if they received reimbursement for BZD drugs for at least 6 months continuously. Prevalence of chronic use at age 65 was estimated from this sample. Incidence of chronic use was estimated by eliminating prevalent users of benzodiazepine at 2010/01/01.

### Modeling assumptions regarding exposure

A subject was considered as exposed as soon as he/she had BZD dispensing for at least 6 consecutive months $(z(a,t) = 1)$. Then he/she remains exposed for any later time *t* and age *a* because we hypothesized that chronic BZD use increased the risk of dementia for the rest of the life. The relative risk of dementia for chronic BZD users was fixed at $\theta_{01} = 1.6$ according to Billioti de Gage et al.^9^. We assumed that the relative risks of death for BZD exposure were identical among demented and non-demented subjects ($\theta_{02} (a) = \theta_{12} (a)$) and constant by 5-years intervals. Based on Mathieu et al.’s results^38^, we took $\theta_{02} (a) = \theta_{12} (a) = 2.45$ for $65 \le a < 70$, $\theta_{02} \left( a \right) = \theta_{12} \left( a \right) = 1.69$ for $70 \le a < 75$, $\theta_{02} \left( a \right) = \theta_{12} \left( a \right) = 1.3$ for $75 \le a < 80$, $\theta_{02} \left( a \right) = \theta_{12} \left( a \right) = 1.1$ for $80 \le a < 85$ and $\theta_{02} \left( a \right) = \theta_{12} \left( a \right) = 1$ for $a \ge 85$. A sensitivity analysis was conducted assuming that BZD use did not increase mortality $\theta_{02} \left( a \right) = \theta_{12} \left( a \right) = 1.$

### Estimations of transition intensities in the illness-death models

The illness-death model stratified by gender was estimated from the Paquid cohort without accounting for BZD consumption. Indeed, in the Paquid cohort, we do not have the information to identify chronic BZD users as defined above: BZD consumption at each visit (every 2 or 3 years) was collected but without information on duration of use. Estimates were obtained by a semi-parametric penalized likelihood approach^39^, using the R package SmoothHazard^40^. This method avoids parametric assumptions on the risk functions and account for both the competing risk of death and the interval censoring of age at dementia between the visit of diagnosis and the previous one. The age-specific relative risk of death for demented subjects, $RR\left( a \right)$, was estimated as the ratio of the estimated mortalities among demented $\alpha_{12} \left( a \right)$ and non-demented subjects $\alpha_{02} \left( a \right)$ without any parametric assumptions.

As several studies have suggested a decreasing calendar time trend for dementia incidence^25^, our main analysis assumed that dementia incidence decreased by 1% each year. This decreasing incidence may reflect the improvement of education or a better care of medical conditions associated with the risk of dementia (hypertension, depression,…). The estimated incidence from Paquid was considered as the incidence for the generations who were 75 years old in 1990 (the median generation in Paquid) and the reduction was 1% each year for the following generations. A sensitivity analysis was conducted assuming a constant age-specific incidence of dementia $\alpha_{01}$.

Then, using the gender-specific estimates of the incidence of dementia, $\alpha_{01} \left( {a,t} \right)$, and of the relative risk of death for demented subjects, $RR\left( a \right)$, as well as the nation-wide projections of age-and gender-specific mortality, we computed the age-, gender- and calendar year-specific projections of mortality for demented $(\alpha_{12} (a,t))$ and non-demented subjects $(\alpha_{02} (a,t))$ by solving a differential equation as described in Wanneveich et al.^31^.

The age-, gender- and calendar year-specific incidence of dementia among non-BZD users $\alpha_{01}^{0} \left( {a,t} \right)$ was computed from the incidence of dementia in the overall population, the age-, gender- and calendar year-specific prevalence of exposure among non-demented subjects ($P_{Z/ND} \left( {a,t} \right)$) and the relative risk $\theta_{01} \left( a \right)$ by solving the following equation:

4 $$ \alpha_{01} \left( {a,t} \right) = P_{Z/ND} \left( {a,t} \right)\alpha_{01}^{0} \left( {a,t} \right)\theta_{01} \left( a \right) + (1 - P_{Z/ND} \left( {a,t} \right))\alpha_{01}^{0} \left( {a,t} \right). $$

Similarly, mortality among exposed and unexposed demented subjects were computed by

5 $$ \alpha_{12} \left( {a,t} \right) = P_{Z/D} \left( {a,t} \right)\alpha_{12}^{0} \left( {a,t} \right)\theta_{12} \left( a \right) + (1 - P_{Z/D} \left( {a,t} \right))\alpha_{12}^{0} \left( {a,t} \right); $$

and mortality among exposed and unexposed non-demented subjects were computed by Eq. (4) replacing $\alpha_{01}^{0} \left( {a,t} \right)$ by $\alpha_{02}^{0} \left( {a,t} \right)$ and $\theta_{01} \left( a \right)$ by $\theta_{02} \left( a \right)$.

### Monte Carlo algorithm for projections in 2040

To provide projections for dementia for subjects older than 65 in 2040, the algorithm was the following:

For each cohort born in year $b$ with $2010 - 105 \le b \le 2040 - 65$ ($1935 \le b \le 1975$), we generated a sample of 10,000 subjects alive and non-demented aged 65 in $b + 65$.

- BZD use at age 65 was generated for each subject according to prevalence of BZD use at 65
- For each subject and each year from $b + 66$ to $b + 105$ or death, we simulated successively the occurrence of BZD use, the death and the onset of dementia according to their respective annual incidences.

Then empirical estimates of the following epidemiological indicators were computed using data from the generated samples with formulas detailed in the Supplementary Online Resource 1: prevalence of exposure (current or past chronic BZD use), prevalence of dementia, life-expectancy without dementia, life-long probability of dementia, mean age at dementia, mean number of years spent with dementia.

A first run of the algorithm was used to estimate only the prevalence of exposure (past or current chronic BZD consumption) that was used to update the computation of the three transition intensities among unexposed subjects ($\alpha_{kj}^{0} ; k = 0, 1; j = 1, 2$) by solving Eqs. (4) and (5). Using these updated values a second run was used to provide central estimations of epidemiological indicators.

Variances of the estimates of epidemiological indicators were computed with 100 runs of the algorithm using 100 different sets of values generated from the posterior distribution of all the parameters estimated in the preliminary steps: $\alpha_{01} \left( a \right)$, $RR\left( a \right)$, BZD chronic use prevalence and BZD chronic use incidence.

All computations were performed separately for women and men for 3 differents scenarios:

- **Scenario 0**: With constant BZD consumption
- **Scenario 1**: Incidence after 65 of chronic BZD use divided by two from 2020
- **Scenario 2**: Incidence after 65 of chronic BZD use considered as null from 2020.

Details for scenarios 1 and 2 are given in Supplementary Online Resource 2. The Monte Carlo algorithm was implemented in the R package MCSPCD available on GitHub.

## Results

Preliminary estimates are presented in the Supplementary Online Resource 2. The estimated incidence of dementia for men and women from the Paquid cohort is displayed in Supplementary Fig. S2 online; Supplementary Fig. S3 online presents the age-and gender-specific relative risks of death for demented versus non-demented subjects and Supplementary Fig. S4 online displays the incidences of chronic use of BZD (6 months straight) by age and gender estimated from EGB. The prevalence of chronic use of BZD at age 65 was estimated at 11.2% (95% CI 9.9–12.6) for men and 18.3 (95% CI 16.7–19.9) for women.

**Figure 1:**
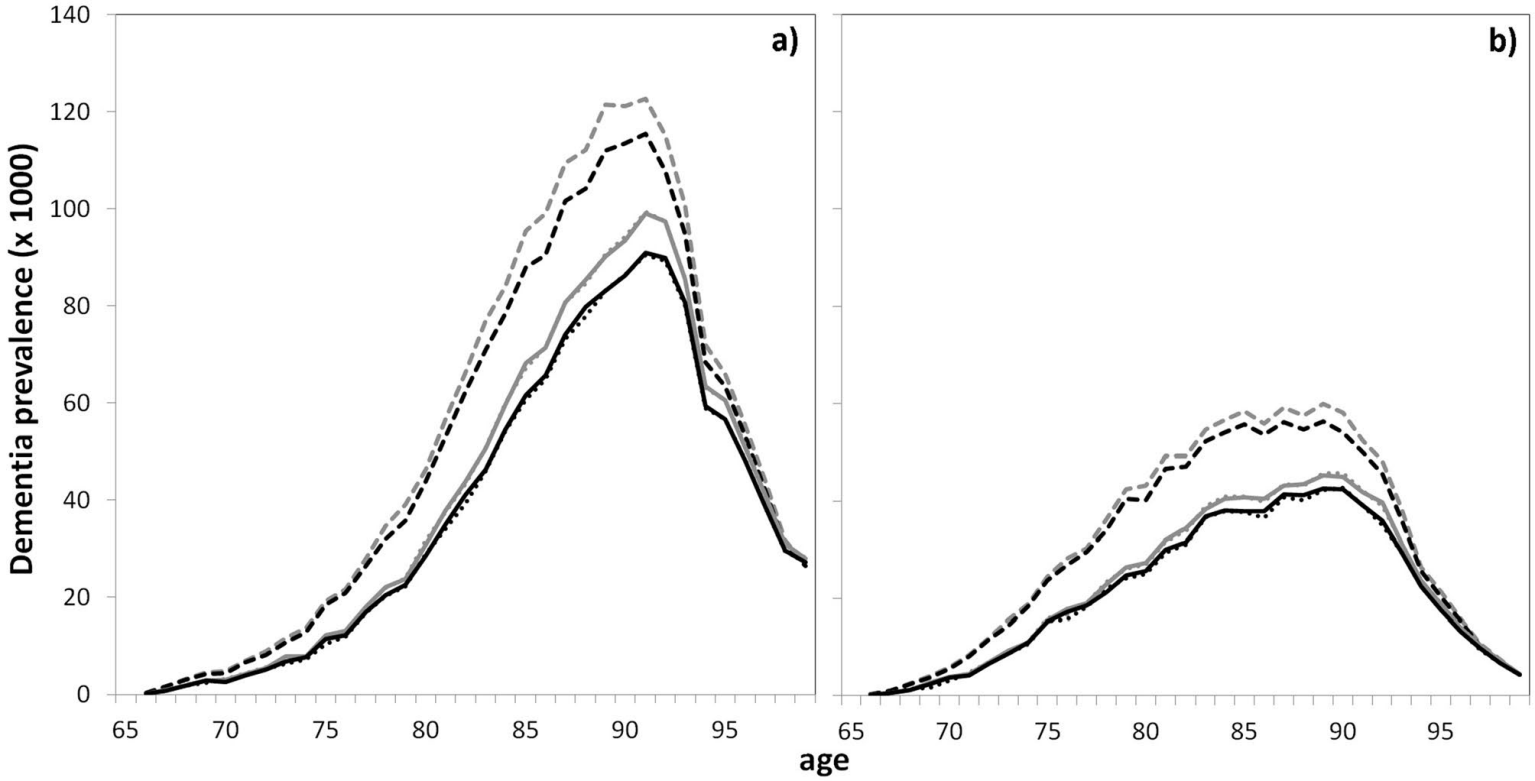
Projections of dementia prevalence by age (in thousands of subjects) in France in 2040 for women (**a**) and men (**b**) for the main analysis and the two sensitivity analyses and for 2 scenarios of BZD use. Grey lines, scenario 0 (constant chronic use of BZD); black lines, scenario 2 (incidence of chronic use of BZD null from 2020); solid lines, main analysis (decreasing dementia incidence and overmortality of exposed subjects); dashed lines, first sensitivity analysis (constant dementia incidence); dotted lines, second sensitivity analysis (no effect of exposure on mortality).

**Figure 2:**
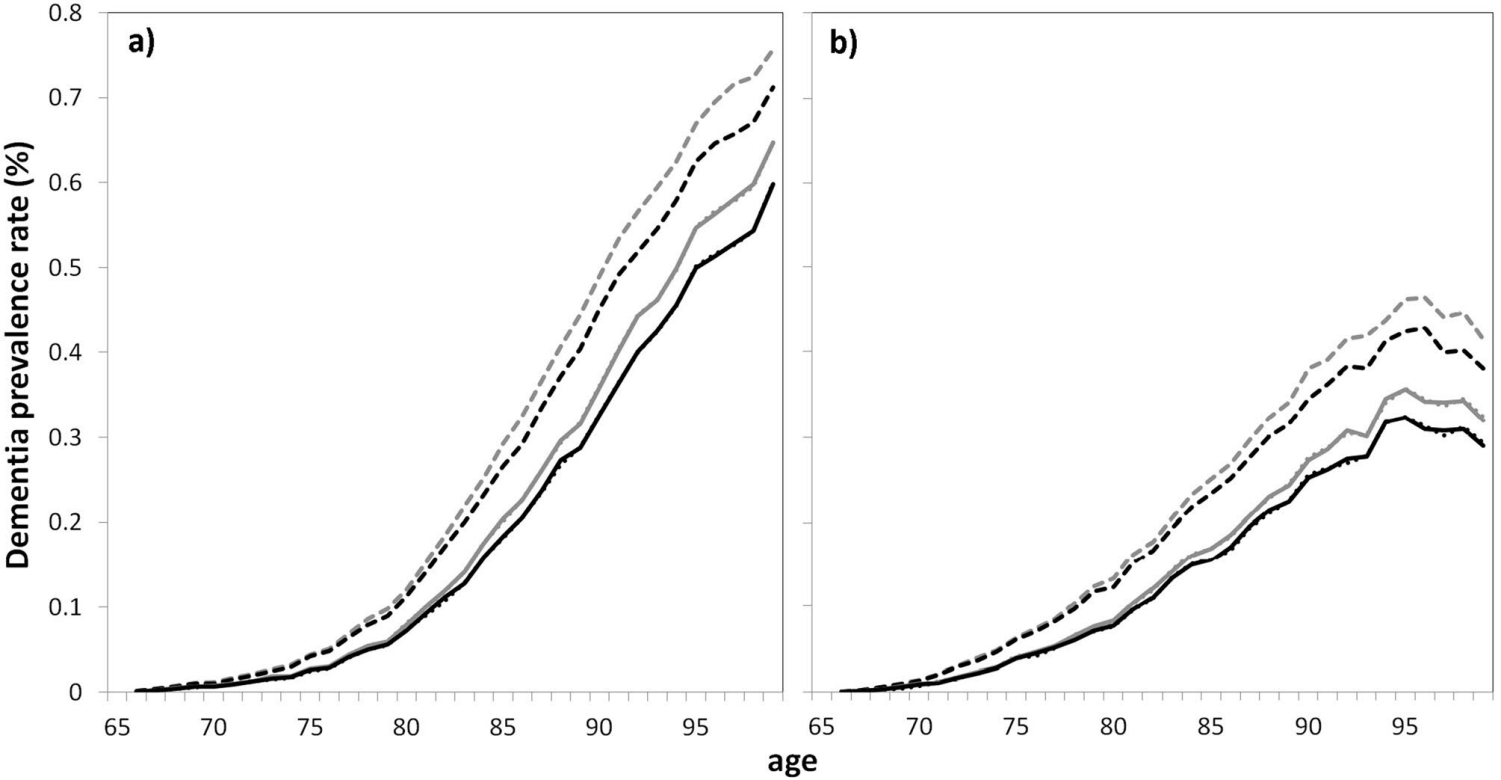
Projections of dementia prevalence rate by age (in %) in France in 2040 for women (**a**) and men (**b**) for the main analysis and the two sensitivity analyses and for 2 scenarios of BZD use. Grey lines, scenario 0 (constant chronic use of BZD); black lines, scenario 2 (incidence of chronic use of BZD null from 2020); solid lines, main analysis (decreasing dementia incidence and overmortality of exposed subjects); dashed lines, first sensitivity analysis (constant dementia incidence); dotted lines, second sensitivity analysis (no effect of exposure on mortality).

**Figure 3:**
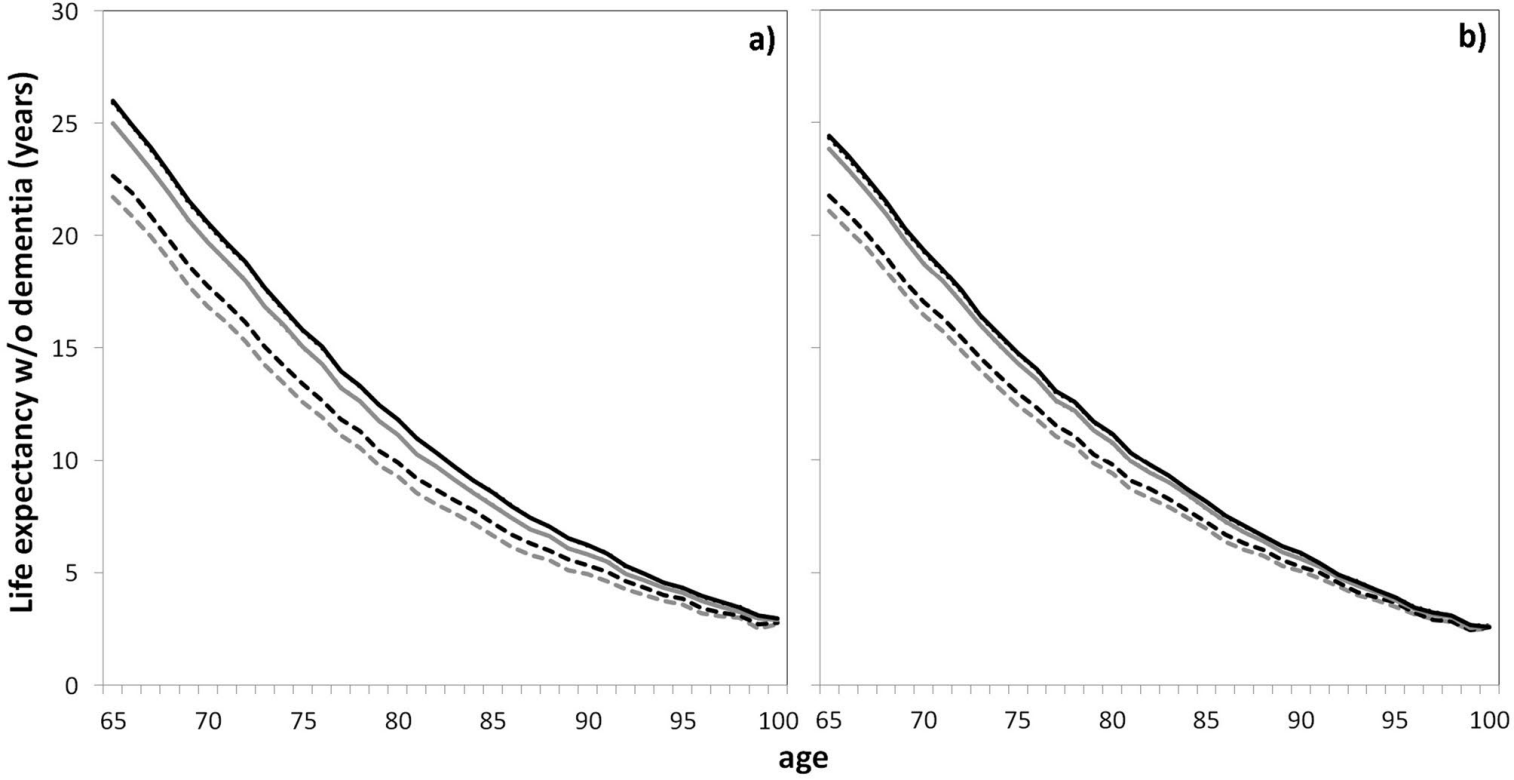
Projections of life expectancies without dementia (in years) at each age from 65 to 100 in France in 2040 for women (**a**) and men (**b**) for the main analysis and the two sensitivity analyses and for 2 scenarios of BZD use. Grey lines, scenario 0 (constant chronic use of BZD); black lines, scenario 2 (incidence of chronic use of BZD null from 2020); solid lines, main analysis (decreasing dementia incidence and overmortality of exposed subjects); dashed lines, first sensitivity analysis (constant dementia incidence); dotted lines, second sensitivity analysis (no effect of exposure on mortality).

Using these estimates, we computed by simulations the projections of the epidemiological indicators for dementia in 2040 for the 3 scenarios of evolution of chronic BZD use. For the main hypotheses (decreasing dementia incidence and overmortality for BZD users), results of scenarios 0 and 2 are compared in Table 1 while scenarios 0 and 1 are compared in Supplementary Table S1 online. Supplementary Figs. S5–S7 online displays the estimated prevalences (number and rates) and the life expectancies with their confidence intervals while Figs. 1, 2 and 3 compare these indicators with those obtained in the sensitivity analyses.

**Table 1:** Projections of dementia burden in France in 2040 for the main hypotheses (decreasing dementia incidence and overmortality of exposed subjects) and for 2 scenarios: constant chronic use of BZD (scenario 0); incidence of chronic use of BZD null from 2020 (scenario 2) and the difference between this 2 scenarios (with 95% confidence intervals).

	Women			Men		
Scenarios	0	2	Difference	0	2	Difference
Prevalence (× 1,000)^a^	1,392 (1,270; 1,515)	1,293 (1,175; 1,411)	− 99 (− 106; − 93)	763 (660; 866)	719 (620; 818)	− 44 (− 50; − 39)
Prevalence rate (%)^a^	12.6 (11.5; 13.6)	11.6 (10.6; 12.6)	− 0.95 (− 1.01; − 0.89)	8.4 (7.3; 9.5)	7.9 (6.8; 8.9)	− 0.54 (− 0.60; − 0.47)
Lifelong probability of dementia at 65 (%)	57.8 (54.2; 61.5)	51.0 (47.6; 54.5)	− 6.8 (− 7.1; − 6.5)	41.3 (36.7; 45.9)	37.0 (32.7; 41.2)	− 4.3 (− 4.8; − 3.8)
Life expectancy w/o dementia at 65 (years)	25.0 (24.7; 25.3)	26.0 (25.7; 26.3)	0.99 (0.93; 1.06)	23.8 (23.5; 24.2)	24.4 (24.0; 24.7)	0.56 (0.50; 0.62)
Average age at dementia (years)	87.8 (87.2; 88.4)	88.0 (87.3; 88.6)	0.13 (0.06; 0.21)	86.3 (85.4; 87.1)	86.2 (85.3; 87.1)	− 0.05 (− 0.15; 0.04)
Mean time spent with dementia (years)^b^	4.5 (4.2; 4.9)	4.0 (3.7; 4.3)	− 0.53 (− 0.56; − 0.49)	2.8 (2.5; 3.2)	2.6 (2.2; 2.9)	− 0.26 (− 0.31; − 0.22)

Abbreviations: w/o, without.

^a^Dementia prevalence between age 65 and 99 (in %).

^b^Mean time spent with dementia for a subject healthy at 65 in 2040 (in years).

Assuming no change in BZD consumption, the predicted number of demented subjects between age 65 and 99 in France in 2040 was 1.39 millions (95% confidence interval (CI) 1.27, 1.52) among women (prevalence rate 12.6%, 95% CI 11.5, 13.6) and 0.76 millions (95% CI 0.66, 0.87) among men (prevalence rate 8.4%, 95% CI 7.3, 9.5). Assuming disappearance of chronic BZD use in 2020 (scenario 2), the prevalence would be reduced by about 99,000 cases (93,000, 106,000) among women and 44,000 cases (39,000, 50,000) among men in 2040. Dividing by two the chronic use of BZD from 2020 (scenario 1), the reduction would be of 46,000 cases (43,000, 50,000) among women and 20,000 cases (17,000, 23,000) among men (Supplementary Table S1 online). For scenario 2, the age-and gender-specific prevalences displayed in Fig. 1 (and in Supplementary Fig. S5 online with the confidence intervals) show that the number of cases would reduce mainly between age 75 and 93 for both gender. However, the prevalence rates would also reduce among the oldests old owing to a slight increase of the population size at these ages (Fig. 2 and Supplementary Fig. S6 online for the confidence intervals). Indeed, the mortality being higher among demented subjects, a decrease in the number of demented cases would lead to a slight increase of the population size among the oldests. Note also that the confidence intervals for the prevalence estimates after age 95 are very large.

Without any change, the probability to develop dementia before death for a woman, respectively a man, 65 years old and not demented in 2040, was estimated at 57.8% (54.2, 61.5), respectively 41.3% (36.7, 45.9). These probabilities would reduce to 51.0% (47.6, 54.5) and 37.0% (32.7, 41.2) without chronic use of BZD from 2020 (scenario 2).

The life expectancy without dementia for a woman aged 65 years in 2040 was estimated at 25.0 years (24.7, 25.3) versus 23.8 years (23.5, 24.2) for a man. The gain with scenario 2 would be 0.99 year (0.93, 1.06) for women and only 0.56 year (0.50, 0.62) for men because their current consumption of BZD is lower. Figure 3 displays the life expectancies without dementia at any age from 65 to 100 for scenarios 0 and 2 (and Supplementary Fig. S6 online with the CI). Whatever the age, the gain for scenario 2 would be larger for women than men.

The estimated mean age at dementia onset was 87.8 (87.2, 88.4) for women and 86.3 (85.4, 87.1) for men, and they would be almost not impacted by a change in BZD consumption.

The mean time spent with dementia between ages 65 and 99 was estimated at 4.5 (4.2, 4.9) years for women and 2.8 (2.5, 3.2) years for men in 2040 and the reduction in case of disappearance of chronic use of BZD would be 0.53 year (0.49, 0.56) for women and 0.26 year (0.22, 0.31) for men.

Results of the two sensitivity analyses are compared with the main results in Figs. 1, 2 and 3 and they are detailed in the Supplementary Tables S2 and S3 online. They showed that the overmortality associated to BZD consumption had a negligible impact on the results because they concerned mainly the youngest where the mortality was low ($\theta_{02} \left( a \right)$ and $\theta_{12} \left( a \right)$ decreased with age and were equal to 1 after age 85). On the other hand, assuming a constant dementia incidence til 2040 instead of a 1% decrease each year, sharply increased all the measures of dementia burden in 2040 but the impact of the scenarios of change in BZD use was similar to the main analysis.

## Discussion

Using a Monte Carlo approach in discrete time, we provided projections of dementia burden in 2040 under various scenarios through several epidemiological indicators that may be useful for public health decision. The prevalence rates of dementia in 2040 were estimated at 12.6% among women and 8.4% among men after age 65. Assuming a causal effect of chronic use of BZD on the risk of dementia, we estimated that a disappearance of this chronic use in 2020 would reduce by 6.6% the number of demented cases in 2040. Halving the rate of chronic use, the reduction in demented cases would be of 3.1%. This impact is not negligible given that this scenario is a reachable objective since it is much less restrictive than the current recommendation for BZD prescription in many countries. However, these results support previous findings^26^ showing that interventions targeting risk factors would have little impact on the age at dementia onset. Thus postponing dementia onset by 1 to 3 years, as previously suggested, would be very difficult^41^.

These projections rely on two main assumptions regarding the effect of BZD on dementia. First, this effect is considered as causal; this is a strong assumption not yet validated. Indeed, previous findings are heterogeneous since some studies do not report any increase risk for BZD users^14,15^. Possible sources of heterogeneity are the definition of BZD users (ever user or minimum length of use), the diagnosis of dementia (clinical, algorithmic or combination of hospital diagnosis or anti-dementia drug use) as well as the design of the studies (prospective or restrospective) and the sample size. However, beyond the existence of the association which is confirmed by several meta-analyses^16–18^, the causal hypothesis is debatable. On one hand, according to Billioti et al.^19^ five of nine Bradford Hill criteria for causality^42^, are fulfilled. These authors consider that the results are consistent enough between studies, the exposure precedes dementia onset, most studies have highlighted a dose response effect and this finding is coherent with known effect of BZD on cognitive function. Finally several plausible mechanisms could explain this effect, the most likely being a decrease of cognitive reserve leading to an earlier onset of dementia^43^. On the other hand, Brandt et al.^44^ conclude that there is not enough evidence to support a causal relationship at that time because of possible indication bias and reverse causality due to the long pre-diagnosis phase of dementia where some symptoms, such as insomnia and anxiety, may be treated by BZD^45^. Second, the increased risk of dementia is assumed to be permanent after a first period of 6 months of BZD use. Given the very long prodromal period before dementia diagnosis, an acute effect of BZD would be unrealistic for this disease and this is coherent with the hypothesized causal mechanism. In addition, subjects treated more than 6 months often remain treated much longer.

The method used account for the possible effect of BZD use on mortality. Nevertheless, changes of treatment in the older adults should be decided cautiously as they may also induce other adverse events that cannot be all evaluated with such a methodology^46^.

The Monte Carlo approach aims at simulating the life of very large samples and then estimating any epidemiological indicators on these samples. Several runs of the algorithm make possible either to compute confidence intervals accounting for the variance of the estimates used for the simulations or to perform sensitivity analysis by choosing a priori alternative values. It is worth to note that our estimates of life expectancy without dementia at age 65 in 2040 can not be interpreted as the standard estimates in demography. The latter is the number of years a woman aged 65 in 2040 would be expected to live without dementia assuming she remains exposed her life-long to the mortality rates of 2040 whereas our estimates account for the expected slight decrease of mortality after 2040.

A great advantage of the Monte Carlo approach in discrete time is its flexibility that makes possible to consider risk factors that may be acquired at any ages and may have reversible effect on the disease of interest. The impact of the discretization of time was evaluated by comparing these results with the continuous time method proposed by Wanneveich et al.^31^. Results were very close. While a non reversible effect after cessation of BZD consumption is plausible, accounting for reversibility could be useful for applications to other risk factors and other diseases. More complex scenarios of changes, such as a progressive decline of the risk factor prevalence, could also be considered. This was not necessary in the framework of BZD and dementia because the magnitude of the effect for scenario 2 was modest. We emphasize that methods for evaluating hypothetical scenarios of intervention always rely on simplifying hypotheses and thus aim at evaluating the magnitude of changes in the disease burden rather than the precise value. Most important is to be able to compare the expected change due to the intervention to the confidence intervals of the estimates, as proposed in this work. Using the R package developed for this work, it is easy to obtain projections for another year or to change some parameters, for instance to evaluate sensitivity of results to the hypothesized value of the relative risk of dementia associated with BZD use.

To conclude, assuming a causal relationship, this work shows that respecting the current recommendation regarding duration of use of BZD could lead to a non negligible decrease of the future burden of dementia.

## Supplementary information

Supplementary information

## Data Availability

The data used to run the simulation program and the datasets generated during the current study are available in the GitHub repository, https://github.com/florianguillet/MCSPCD.
