# Supplementary material for: Impact of benzodiazepine consumption reduction on future burden of dementia
Source: Sci Rep. 2020 Sep 4;10:14666. doi: 10.1038/s41598-020-71482-0 (PMC7474098; doi:10.1038/s41598-020-71482-0)
Supplement: Supplementary file 1 — Supplementary information [file 41598_2020_71482_MOESM1_ESM.docx]

**SUPPLEMENTARY** **INFORMATIONS**

**For the manuscript**

**Impact of Benzodiazepine Consumption Reduction on Future Burden of Dementia**

Hélène Jacqmin-Gadda, Florian Guillet, Clément Mathieu, Catherine Helmer,

Antoine Pariente, Pierre Joly

**Online Resource 1: Estimators of epidemiological indicators**

Let us denote $n_{i}\left( a,t \right)$ the number of subjects in state i (i=0,1 for healthy and demented) at age $a$ in year $t$ in the simulated cohorts, $n_{i}\left( a,t | z(a,t)=1 \right)$ the number of subjects in state i at age $a$ in year $t$ among subjects currently exposed and $n_{ij}(a,t)$ the number of subjects in state $i$ at age $a$ in year $t$ and in state $j$ at age $a+1$ in year $t+1$ Note that $n_{0}(a,t)+n_{1}(a,t)$ is the total number of subjects of age a alive in year t. With these notations, the various epidemiological indicators were empirically estimated from the simulated samples with the following formulas:

- Prevalence rate of exposure at age $a$ in year $t$:

$$P_{Z}(a,t)=\frac{n_{0}(a,t\mid z(a,t)=1)+n_{1}(a,t\mid z(a,t)=1)}{n_{0}(a,t)+n_{1}(a,t)}$$

Among non demented subjects:

$$P_{Z/ND}(a,t)=\frac{n_{0}(a,t\mid z(a,t)=1)}{n_{0}(a,t)}$$

Among demented subjects:

$$P_{Z/D}(a,t)=\frac{n_{1}(a,t\mid z(a,t)=1)}{n_{1}(a,t)}$$

- Prevalence rate of dementia at age $a$ in year $t$:

$$P_{1}(a,t)=\frac{n_{1}(a,t)}{n_{0}(a,t)+n_{1}(a,t)}$$

- Proportion of healthy subjects at age $a$ in year t

$$P_{0}\left( a,t \right)=1- P_{1}\left( a,t \right)$$

- Number of cases of dementia in year $t$for the total French population (cumulated over all considered ages) :

$$Prev\left( t \right)=\sum_{a=65}^{99} N_{1}\left( a,t \right)= \sum_{a=65}^{99} \nu\left( 65, t-a+65 \right)P_{01}\left( a,t \right)$$

Where $N_{1}\left( a,t \right)$is the number of demented subjects of age a the year t in the French population, $\nu(65,t-a +65)$ is the size of the population at risk at age 65 for the year $t-a+65$ (surviving subjects at age 65 from the birth cohort $t-a$) provided by the INSEE projection and $P_{01}\left( a,t \right)$ .is the probability for a subject alive at age 65 to be alive and demented at age a and year t, which is estimated by:

$$P_{01}\left( a,t \right)= \frac{n_{1}(a,t)}{n_{0}(65,t-a+65)}$$

Note that all subjects are assumed to be free of dementia at age 65.

- Life-expectancy without dementia for a subject healthy at age $a$ in year $t$:

$$LE_{00}(a,t)=0.5+\sum_{u=a}^{104} \frac{n_{00}(u,t-a+u)}{n_{0}(a,t)}$$

- Overall life-expectancy for a non-demented subject at age 65 in year $t$:

$$LE_{0.}(65,t)=0.5+\sum_{u=a}^{104} \frac{n_{00}(u,t \_65 +)+n_{01}(u,t-65+u )+n_{11}(u,t-65+u )}{n_{0}(65,t)}$$

- Mean number of years spent with dementia for a subjects healthy at age 65 in year $t$:

$$T_{11}(65,t)=LE_{0.}(65,t)-LE_{00}(65,t)$$

- Life-long probability of dementia for a healthy subject of age $a$ in year $t$:

$$F_{01}(a,t)=\sum_{u=a}^{99} \frac{n_{01}(u,t+u-a)}{n_{0}(a,t)}$$

- Average age at dementia onset for subjects healthy at age 65 in year $t$:

$$AV_{01}(t)=\frac{\sum_{u=65}^{99} u.n_{01}(u,t-65+u)}{\sum_{u=65}^{99} n_{01}(u,t-65 +u)}$$

**Online Resource 2: details for scenarios 1 and 2**

Note that a reduction in incidence of BZD use from 2020 induces a decreasing prevalence of use at age 65 according to years from 2020 to 2040. For instance, for scenario 2, the prevalence at age 65 for the year 2020+x is the prevalence at age 65-x for 2020 because there is no new chronic BZD users from 2020. The prevalence in 2020 for ages 45 to 64 were computed assuming that the prevalence at age 45 was half the prevalence at age 65 (as suggested by Richard et al. (1)) and that the incidence was constant between age 45 and 65. For scenario 1, this incidence was divided by 2.


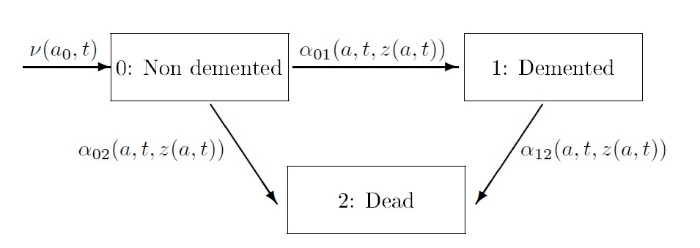


Figure S1. The illness-death model : $\boldsymbol{\alpha}_{\mathbf{01}}$ is the incidence of dementia, $\boldsymbol{\alpha}_{\mathbf{02}}$ and $\boldsymbol{\alpha}_{\mathbf{12}}$ are the mortality of healthy and demented subjects, $\mathbf{a}$ is the age, $\mathbf{t}$ is the calendar year and $\mathbf{z(a,t)}$ is the time and age dependent binary exposure, $\boldsymbol{\nu(}\boldsymbol{a}_{\boldsymbol{0}}\mathbf{,t)}$ is the population size at age $\boldsymbol{a}_{\boldsymbol{0}}$ and year $\mathbf{t}$.


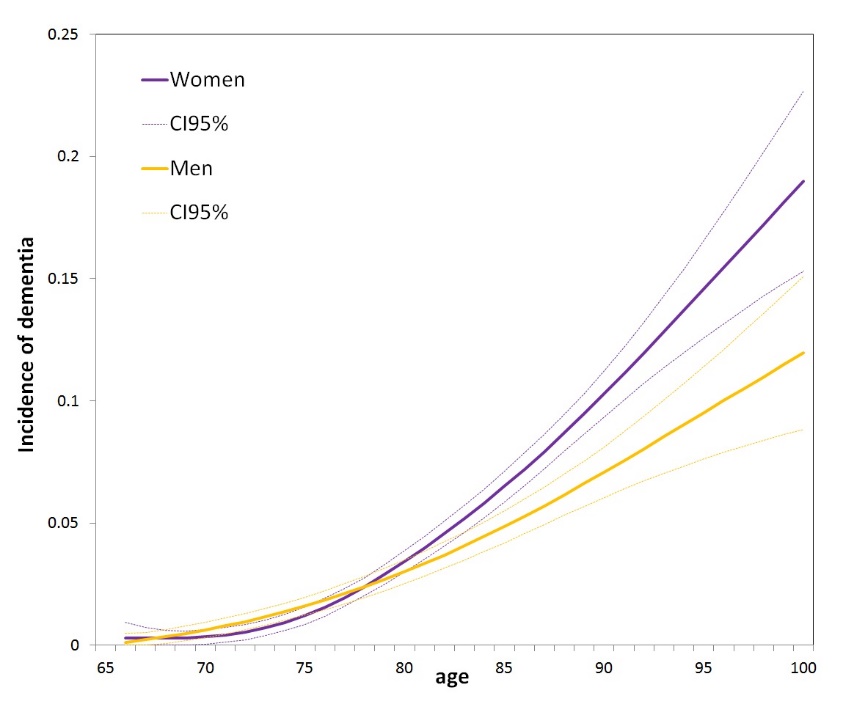


Figure S2. Estimated incidence of dementia age and gender: PAQUID France, 1989-2015.


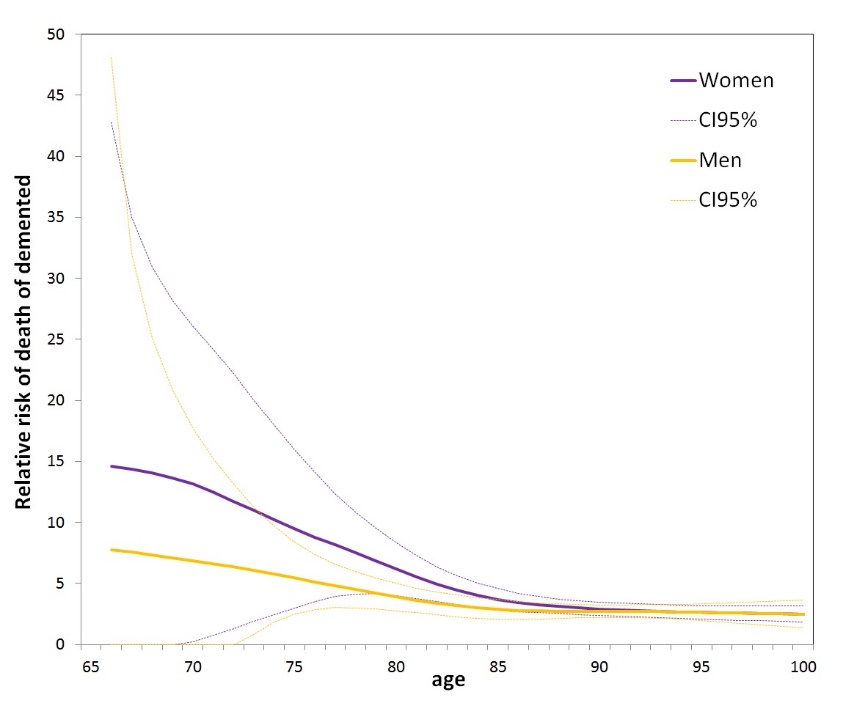


Figure S3. Age-and gender-specific relative risk of death for demented versus non demented subjects: PAQUID France, 1989-2015.


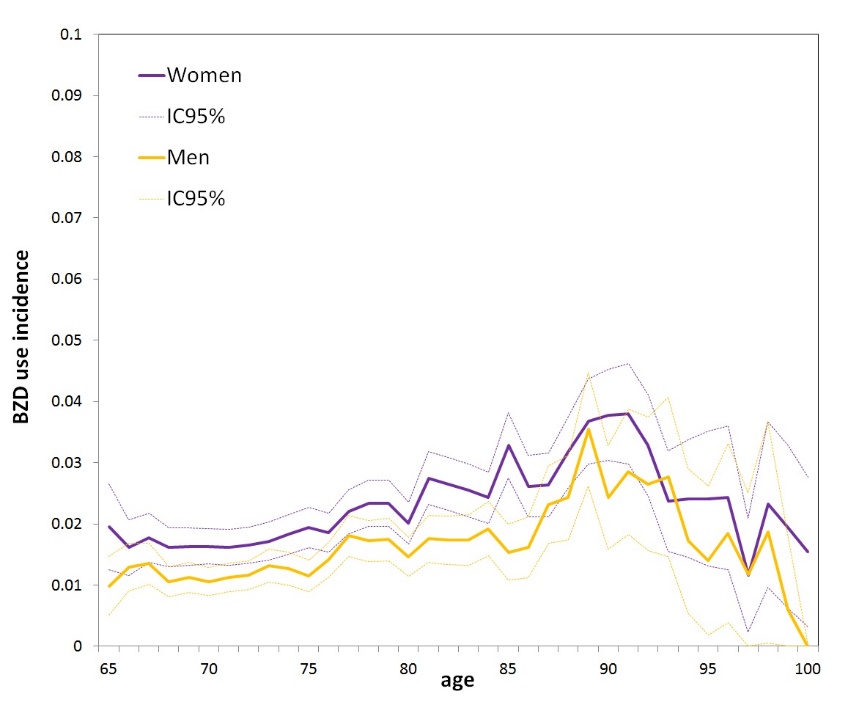


Figure S4. Age-and gender-specific incidence of chronic use of BZD: EGB France, 2010-2015.

Table S1. Projections of dementia burden in France in 2040 for the main hypotheses (decreasing dementia incidence and overmortality of exposed subjects) and for 2 scenarios: constant chronic use of BZD (scenario 0); incidence of chronic use of BZD divided by two from 2020 (scenario 1) and the difference between this 2 scenarios (with 95% confidence intervals).

|  | Women | | | Men | | |
| --- | --- | --- | --- | --- | --- | --- |
| Scenarios | 0 | 1 | Difference | 0 | 1 | Difference |
| Prevalence  (x 1000) ^a^ | 1,392  (1,270; 1,515) | 1,346  (1,226; 1,466) | -46  (-50; -43) | 763  (660; 866) | 743  (642; 844) | -20  (-23; -17) |
| Prevalence rate (%)^a^ | 12.6  (11.5; 13.6) | 12.1  (11.1; 13.2) | -0.45  (-0.48; -0.41) | 8.4  (7.3; 9.5) | 8.2  (7.1; 9.2) | -0.25  (-0.28; -0.21) |
| Lifelong probability of dementia at 65 (%) | 57.8  (54.2; 61.5) | 54.9  (51.4; 58.4) | -2.9  (-3.1; -2.7) | 41.3  (36.7; 45.9) | 39.2  (34.8; 43.7) | -2.1  (-2.3; -1.8) |
| Life expectancy w/o dementia at 65 (years) | 25.0  (24.7; 25.3) | 25.4  (25.1; 25.8) | 0.46  (0.43; 0.49) | 23.8  (23.5; 24.2) | 24.1  (23.8; 24.4) | 0.27  (0.24; 0.31) |
| Average age at dementia (years) | 87.8  (87.2; 88.4) | 88.0  (87.4; 88.6) | 0.13  (0.09; 0.18) | 86.3  (85.4; 87.1) | 86.3  (85.4; 87.2) | 0.03  (-0.03; 0.09) |
| Mean time spent with dementia (years) ^b^ | 4.5  (4.2; 4.9) | 4.3  (4.0; 4.6) | -0.24  (-0.26; -0.22) | 2.8  (2.5; 3.2) | 2.7  (2.3; 3.0) | -0.13  (-0.16; -0.11) |

Abbreviations: w/o, without.

^a^Dementia prevalence between age 65 and 99 (in %)

^b^Mean time spent with dementia for a subject healthy at 65 in 2040 (in years)

Table S2. First sensitivity analysis (constant dementia incidence and overmortality of exposed subjects). Projections of dementia burden in France in 2040 for 3 scenarios: constant chronic use of BZD (scenario 0); division by two of the incidence of chronic use of BZD from 2020 (scenario 1); incidence of chronic use of BZD null from 2020 (scenario 2) and the difference between scenarios (with 95% confidence intervals).

|  | Women | | | Men | | |
| --- | --- | --- | --- | --- | --- | --- |
| Scenarios | 0 | 1 | Difference | 0 | 1 | Difference |
| Prevalence  (x 1000) ^a^ | 1,821  (1,657; 1,986) | 1,770  (1,608; 1,932) | -51  (-54; -47) | 1,057  (915; 1,199) | 1,033  (894; 1,172) | -24  (-28; -20) |
| Prevalence rate (%)^a^ | 16.8  (15.3; 18.3) | 16.3  (14.8; 17.7) | -0.51  (-0.55; -0.48) | 11.9  (10.3; 13.5) | 11.6  (10.1; 13.1) | -0.31  (-0.36; -0.27) |
| Lifelong probability of dementia at 65 (%) | 76.4  (73.0; 79.9) | 74.5  (70.9; 78.1) | -1.9  (-2.2; -1.6) | 60.5  (55.3; 65.7) | 58.5  (53.4; 63.6) | -2.0  (-2.2; -1.8) |
| Life expectancy w/o dementia at 65 (years) | 21.7  (21.2; 22.2) | 22.2  (21.7; 22.6) | 0.46  (0.43; 0.50) | 21.1  (20.6; 21.6) | 21.4  (20.9; 21.9) | 0.32  (0.29; 0.36) |
| Average age at dementia (years) | 86.0  (85.3; 86.6) | 86.2  (85.6; 86.9) | 0.25  (0.20; 0.30) | 84.8  (84.0; 85.7) | 85.0  (84.1; 85.9) | 0.15  (0.11; 0.19) |
| Mean time spent with dementia (years) ^b^ | 6.7  (6.2; 7.2) | 6.4  (5.9; 6.9) | -0.25  (-0.27; -0.22) | 4.5  (4.0; 5.1) | 4.4  (3.9; 4.9) | -0.16  (-0.18; -0.14) |
| Scenarios | 0 | 2 | Difference | 0 | 2 | Difference |
| Prevalence  (x 1000) ^a^ | 1,821  (1,657; 1,986) | 1,705  (1,546; 1,865) | -116  (-123; -109) | 1,057  (915; 1,199) | 1,005  (868; 1,142) | -52  (-58; -46) |
| Prevalence rate (%)^a^ | 16.8  (15.3; 18.3) | 15.6  (14.2; 17.0) | -1.15  (-1.22; -1.09) | 11.9  (10.3; 13.5) | 11.2  (9.7; 12.7) | -0.66  (-0.74; -0.58) |
| Lifelong probability of dementia at 65 (%) | 76.4  (73.0; 79.9) | 71.8  (68.0; 75.6) | -4.6  (-5.2; -4.0) | 60.5  (55.3; 65.7) | 56.1  (51.1; 61.2) | -4.4  (-4.7; -4.0) |
| Life expectancy w/o dementia at 65 (years) | 21.7  (21.2; 22.2) | 22.7  (22.2; 23.1) | 0.97  (0.91; 1.03) | 21.1  (20.6; 21.6) | 21.8  (21.3; 22.3) | 0.68  (0.63; 0.74) |
| Average age at dementia (years) | 86.0  (85.3; 86.6) | 86.5  (85.8; 87.1) | 0.47  (0.39; 0.55) | 84.8  (84.0; 85.7) | 85.1  (84.2; 86.0) | 0.22  (0.14; 0.30) |
| Mean time spent with dementia (years) ^b^ | 6.7  (6.2; 7.2) | 6.2  (5.7; 6.6) | -0.51  (-0.56; -0.47) | 4.5  (4.0; 5.1) | 4.2  (3.7; 4.7) | -0.35  (-0.38; -0.32) |

Abbreviations: w/o, without.

^a^Dementia prevalence between age 65 and 99 (in %)

^b^Mean time spent with dementia for a subject healthy at 65 in 2040 (in years)

Table S3. Second sensitivity analysis (decreasing dementia incidence and no effect of exposure on mortality). Projections of dementia burden in France in 2040 for 3 scenarios: constant chronic use of BZD (scenario 0); division by two of the incidence of chronic use of BZD from 2020 (scenario 1); incidence of chronic use of BZD null from 2020 (scenario 2) and the difference between scenarios (with 95% confidence intervals).

|  | Women | | | Men | | |
| --- | --- | --- | --- | --- | --- | --- |
| Scenarios | 0 | 1 | Difference | 0 | 1 | Difference |
| Prevalence  (x 1000)^a^ | 1 391  (1 271, 1 511) | 1 339  (1 223, 1 456) | -52  (-56, -48) | 762  (661, 863) | 738  (639, 837) | -24  (-27, -21) |
| Prevalence rate (%)^a^ | 12.6  (11.5, 13.6) | 12.1  (11.1, 13.1) | -0.48  (-0.52, -0.45) | 8.4  (7.3, 9.5) | 8.1  (7.1, 9.2) | -0.27  (-0.30, -0.24) |
| Lifelong probability of dementia (%) | 57.8  (54.2, 61.5) | 54.8  (51.3, 58.3) | -3.0  (-3.2, -2.8) | 41.1  (36.6, 45.6) | 39.0  (34.6, 43.4) | -2.1  (-2.3, -1.9) |
| Life expectancy w/o dementia at 65 (years) | 25.0  (24.7, 25.3) | 25.4  (25.1, 25.7) | 0.42  (0.39, 0.45) | 23.8  (23.5, 24.2) | 24.1  (23.7, 24.4) | 0.23  (0.20, 0.26) |
| Average age at dementia (years) | 87.8  (87.2, 88.4) | 88.0  (87.3, 88.6) | 0.13  (0.09, 0.17) | 86.3  (85.4, 87.1) | 86.3  (85.4, 87.2) | 0.01  (-0.06, 0.07) |
| Mean time spent with dementia (years) ^b^ | 4.5  (4.2, 4.9) | 4.3  (3.9, 4.6) | -0.26  (-0.28, -0.24) | 2.8  (2.5, 3.2) | 2.7  (2.3, 3.0) | -0.14  (-0.17, -0.12) |
| Scenarios | 0 | 2 | Difference | 0 | 2 | Difference |
| Prevalence  (x 1000) ^a^ | 1 391  (1 271, 1 511) | 1 281  (1 168, 1 395) | -110  (-117, -103) | 762  (661, 863) | 710  (615, 806) | -52  (-57, -46) |
| Prevalence rate (%)^a^ | 12.6  (11.5, 13.6) | 11.5  (10.6, 12.5) | -1.02  (-1.09, -0.95) | 8.4  (7.3, 9.5) | 7.8  (6.8, 8.8) | -0.58  (-0.65, -0.52) |
| Lifelong probability of dementia at 65 (%) | 57.8  (54.2, 61.5) | 50.8  (47.4, 54.3) | -7.0  (-7.4, -6.6) | 41.1  (36.6, 45.6) | 36.7  (32.4, 40.9) | -4.4  (-4.9, -4.0) |
| Life expectancy w/o dementia at 65 (years) | 25.0  (24.7, 25.3) | 25.9  (25.6, 26.2) | 0.91  (0.85, 0.98) | 23.8  (23.5, 24.2) | 24.3  (24.0, 24.6) | 0.47  (0.41, 0.53) |
| Average age at dementia (years) | 87.8  (87.2, 88.4) | 88.0  (87.3, 88.6) | 0.15  (0.08, 0.21) | 86.3  (85.4, 87.1) | 86.2  (85.2, 87.1) | -0.10  (-0.20, 0.00) |
| Mean time spent with dementia (years)^b^ | 4.5  (4.2, 4.9) | 4.0  (3.7, 4.3) | -0.57  (-0.61, -0.53) | 2.8  (2.5, 3.2) | 2.5  (2.2, 2.9) | -0.28  (-0.32, -0.25) |

Abbreviations: w/o, without.

^a^Dementia prevalence between age 65 and 99 (in %)

^b^Mean time spent with dementia for a subject healthy at 65 in 2040 (in years)


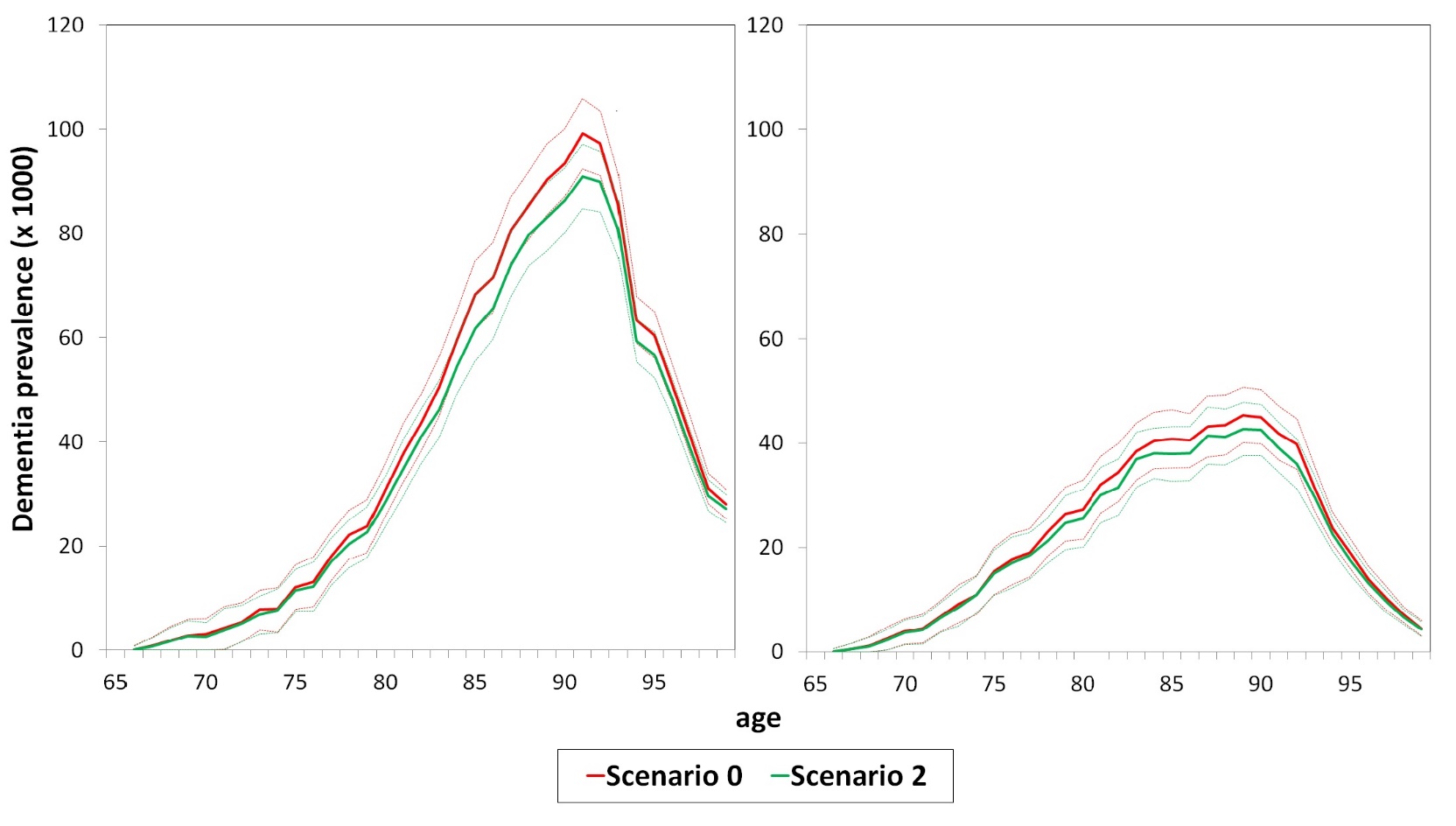


Figure S5. Projections of dementia prevalence by age (in thousands of subjects) in France in 2040 for women (left) and men (right) with 95% confidence intervals assuming decreasing dementia incidence and overmortality of exposed subjects (main hypotheses) for 2 scenarios: constant chronic use of BZD (scenario 0); incidence of chronic use of BZD null from 2020 (scenario 2).


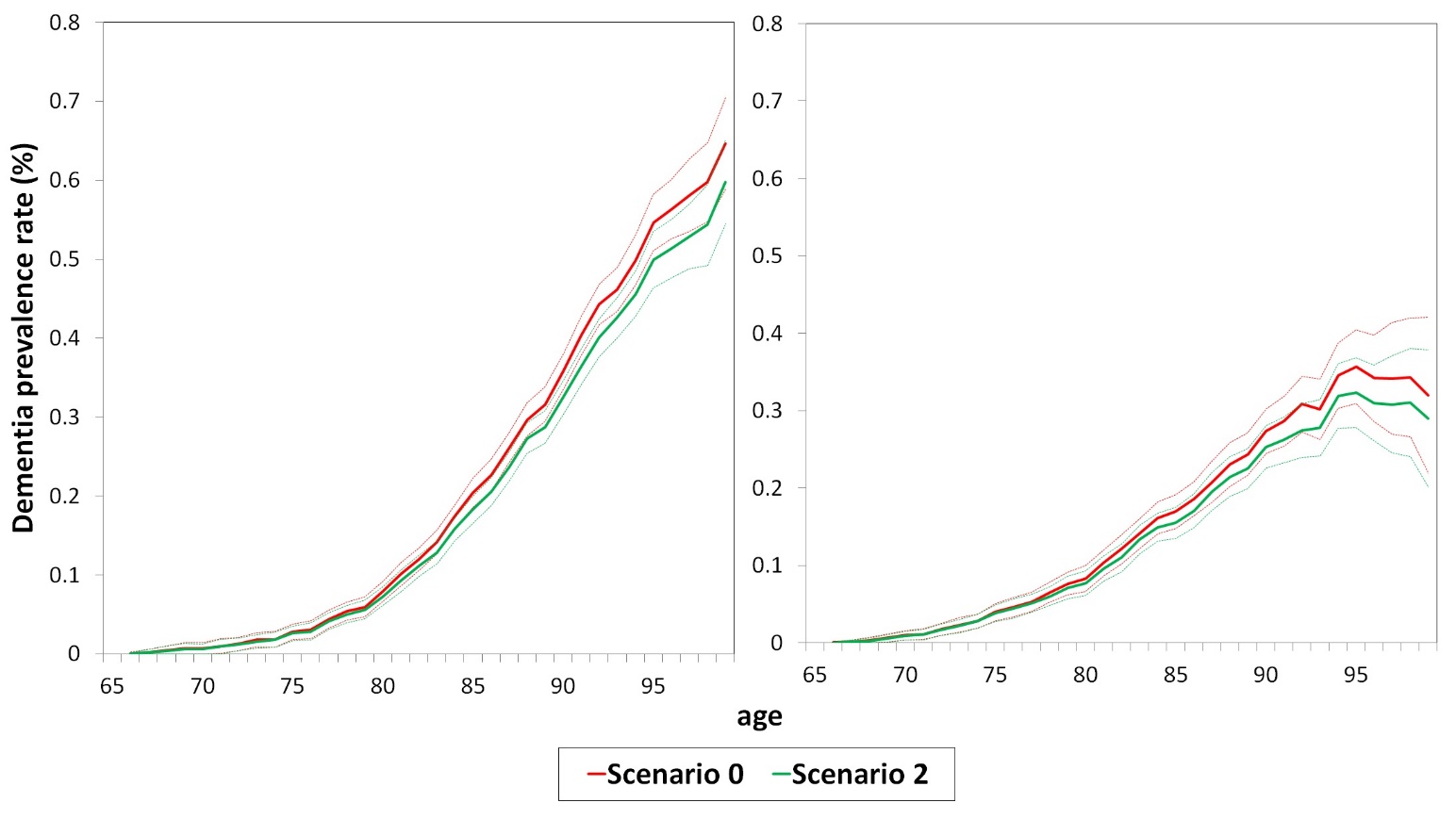


Figure S6. Projections of dementia prevalence rates by age (in %) in France in 2040 for women (left) and men (right) with 95% confidence intervals assuming decreasing dementia incidence and overmortality of exposed subjects (main hypotheses) for 2 scenarios: constant chronic use of BZD (scenario 0); incidence of chronic use of BZD null from 2020 (scenario 2).


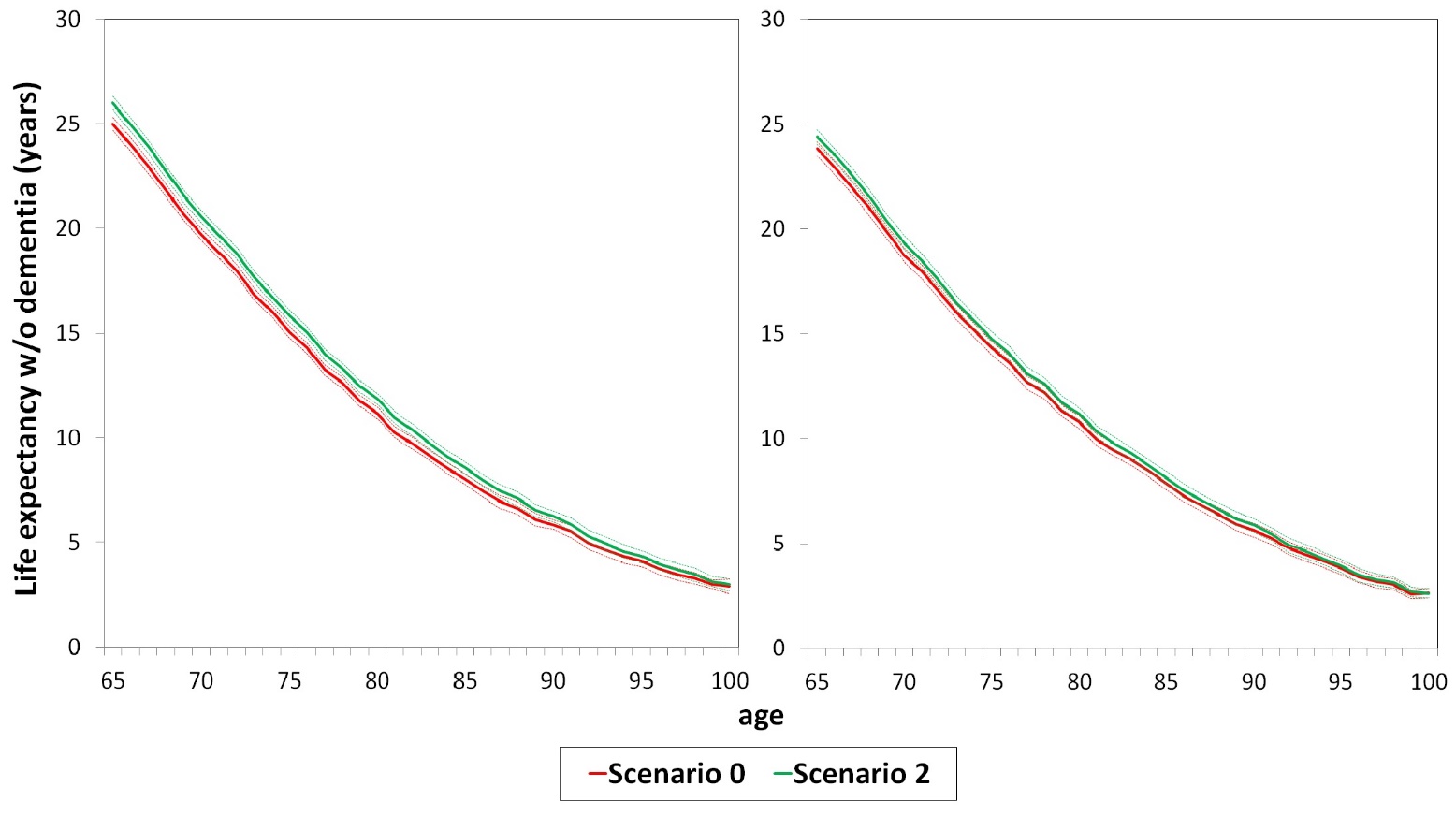


Figure S7. Projections of life expectancies without dementia (in years) at each age from 65 to 100 in France in 2040 for women (left) and men (right) with 95% confidence intervals assuming decreasing dementia incidence and overmortality of exposed subjects (main hypotheses) for 2 scenarios: constant chronic use of BZD (scenario 0); incidence of chronic use of BZD null from 2020 (scenario 2).

# Online References

1. Richard N, Benard A, Billioti de Gage S, et al. *État des lieux de la consommation des benzodiazépines en France.*ANSM;2017.
